# Spatiotemporal processing of somatosensory stimuli in schizotypy

**DOI:** 10.1038/srep38735

**Published:** 2016-12-09

**Authors:** Francesca Ferri, Ettore Ambrosini, Marcello Costantini

**Affiliations:** 1 Centre for Brain Science, Department of Psychology, University of Essex, Colchester, UK; 2 Department of Psychology, University of Padua, Padua, Italy; 3 Laboratory of Neuropsychology and Cognitive Neuroscience, Department of Neuroscience and Imaging, University G. d’Annunzio & Institute for Advanced Biomedical Technologies - ITAB, Foundation University G. d’Annunzio, Chieti, Italy

## Abstract

Unusual interaction behaviors and perceptual aberrations, like those occurring in schizotypy and schizophrenia, may in part originate from impaired remapping of environmental stimuli in the body space. Such remapping is contributed by the integration of tactile and proprioceptive information about current body posture with other exteroceptive spatial information. Surprisingly, no study has investigated whether alterations in such remapping occur in psychosis-prone individuals. Four hundred eleven students were screened with respect to schizotypal traits using the Schizotypal Personality Questionnaire. A subgroup of them, classified as low, moderate, and high schizotypes were to perform a temporal order judgment task of tactile stimuli delivered on their hands, with both uncrossed and crossed arms. Results revealed marked differences in touch remapping in the high schizotypes as compared to low and moderate schizotypes. For the first time here we reveal that the remapping of environmental stimuli in the body space, an essential function to demarcate the boundaries between self and external world, is altered in schizotypy. Results are discussed in relation to recent models of ‘self-disorders’ as due to perceptual incoherence.

Daily interactions with people and objects involve locating stimuli in the environment, and relative to ourselves. For instance, to respond to a touch, being an insect pricking your leg or a friend patting you on the back, it is essential to localize the tactile stimulus in space. The computation of such spatial localization is critically dependent upon coordinate transformation between several reference frames, including body-centered space and external space^1^,^2^, as well as the integration of tactile and proprioceptive information about current body posture with other exteroceptive spatial information^3^. Consequently, impaired processing of tactile and proprioceptive cues from the body, as well as deficits in integrating multisensory information from the body and the environment, might undermine the ability to remap touch in the external space, ultimately leading to unusual perceptual aberrations and interaction behaviors. This can be the case in schizotypy and schizophrenia.

Indeed, empirical evidence suggests that increased levels of schizotypy-related deviance (e.g. abnormal perceptions, odd beliefs and eccentric behavior), as well as schizophrenia symptoms, are associated with tactile and proprioceptive dysfunctions^4^,^5^. In addition, a growing body of research makes the case that the loss of coherent perception of one’s body and the external world in schizotypy and schizophrenia might reflect abnormal multisensory processing^6^,^7^,^8^,^9^.

Schizotypy is thought to reflect the subclinical expression of the symptoms of schizophrenia in the general population and to constitute a dynamic continuum ranging from personality variation to psychosis^10^,^11^,^12^. Even if most schizotypes are not expected to develop psychosis, schizotypy is associated with heightened risk for the development of psychotic disorders^13^. Indeed, substantial overlapping has been found between schizotypy and schizophrenia not only in terms of etiological factors - at the genetic, biological, and psychosocial levels^14^ – but also concerning a wide range of perceptual, cognitive, and motor impairments^15^. However, the study of perceptual and multisensory integration dysfunction in schizotypy offers the advantage of preventing the psychological variables from being confounded by compensatory strategies, severity, distress, comorbidity, and therapy.

With respect to proprioceptive processing, it is worth to note here that a “spatial-kinesthetic-vestibular dysfunction” (i.e., proprioceptive deficit) was designated as a cardinal aspect of schizotypy since its first significant model was proposed^16^. Recent empirical evidence^4^ supports this idea showing that a worse performance on the weight discrimination task is associated with higher positive (cognitive-perceptual) and negative (interpersonal) factors of the Schizotypal Personality Questionnaire (SPQ)^17^. Similarly, with respect to tactile processing, higher positive schizotypy has been associated to decreased touch sensitivity – i.e., higher two-point discrimination threshold^4^,^5^.

Abnormal integration of multisensory cues from the body (i.e. tactile and proprioceptive) with those from the environment (i.e. visual) has been revealed in schizotypy by the rubber hand illusion (RHI). In general, during RHI participants develop the feeling that an artificial hand belongs to themselves under the condition that their own hand and the artificial hand are touched synchronously, and that they are in the same position^18^,^19^,^20^. Normally, as mismatch increases beyond a critical temporal or spatial value, the integration of multisensory cues suddenly breaks down^21^,^22^. Differently, schizotypes^6^,^8^ show increased proneness to the illusion also during asynchronous stimulation^8^. This suggests that the integration of tactile and external visual information is altered. A possible explanation is that schizotypes assign more weight to visual external cues, than to tactile and proprioceptive information from the body, also given the potential weakness of the latter (see above).

In sum, there is clear and convincing evidence of abnormal processing of tactile and proprioceptive information in schizotypy, together with altered multisensory integration of cues from the body and the environment. Surprisingly, however, no investigation of a possible consequent deficit in the ability to remap touch in the external space has been conducted so far. Thus, we still do not know whether there are abnormalities of touch remapping in schizotypy that significantly exceed the contribution of tactile and proprioceptive deficits to perceptual aberrations and unusual interaction behavior.

One reliable way to test this hypothesis consists in performing a temporal order judgment (TOJ) task while the hands are either uncrossed or crossed over the body midline^23^,^24^. In a typical TOJ task, participants perform judgments about the order of two tactile stimuli presented one after the other to the left and right hands. When the hands are uncrossed, the task can be solved using tactile and proprioceptive information about the body posture, encoded on the basis of a body-centered reference frame^1^,^2^. However, when the hands are crossed, the same information must be integrated with a representation of visual space and remapped on the basis of an external reference frame^1^,^2^.

In this study, we asked participants with high, moderate, and low schizotypy to perform the TOJ task, with both uncrossed and crossed hands. Drawing from the evidence reported above, we expected stronger cross-hand effect to be i) associated to higher schizotypy, and ii) only partially explained by eventual differences already present between the groups during the uncrossed-hand task.

## Method

### Participants

Four hundred eleven undergraduate and postgraduate students, recruited via mailing lists at the University “G. d’Annunzio” of Chieti-Pescara, were screened with respect to schizotypal traits using the SPQ^17^. The distribution of scores (see Supplementary Figure S1 and Supplementary Table T1) was divided into quintile, with fifth quintile representing the students rated as high schizotypes, the first quintile representing the students rated as low schizotypes, and the third quintile representing the student rated as moderate schizotypes. Students falling within the second and fourth quintiles were not invited to perform the task. Sixteen students falling within each of the quintiles used (first, third and fifth) were recruited for the study, for a total of forty-eight participants. An a-priori sensitivity power analysis (G*Power^25^) revealed that our sample is large enough to provide ≥80% power to detect between-groups differences in TOJ performance corresponding to an effect size f = 0.46. This effect size estimate was chosen based on a recent study^26^ showing that psychosis-proneness in healthy volunteers significantly predicted susceptibility to experiencing the rubber hand illusion (which rely on multisensory integration processes) with an effect size f = 0.465. Moreover, the effect size estimate we used in the power analysis is lower than those observed in existing studies showing very large effect size estimates (i.e., f ≥ 0.475) for altered multisensory integration (e.g.:^27^: f = 0.71;^28^: f = 0.60) and temporal order judgment performance^29^: (fs > 0.49) in SCZ patients. All participants were right-handed and had normal tactile sensitivity as self-reported. Participants did not report suffering from neuropathy, which might affect tactile sensitivity. Age, education, and gender were matched across groups. In Table 1 demographic details are reported. The study was approved by the local ethical committee (University “G. d’Annunzio” of Chieti-Pescara) and it was performed in compliance with Declaration of Helsinki’s ethical principles (World Medical Association, 1991). All the participants signed an Informed Consent form.

### Procedure

Participants sat at a table with their hands lying palm down on the table surface. In one condition (crossed condition), they were asked to hold their arms crossed over the wrist, whereas in the other condition (uncrossed condition), they were asked to hold their arms uncrossed. In the crossed condition, the two possible relative positions of the arms (right over left and vice versa) were counterbalanced across participants and their order was assigned randomly to each participants. The distance between the hands was kept constant in the crossed and uncrossed conditions.

Two tactile stimuli were presented in rapid succession, one to each hand. The time interval between the two stimuli (i.e., the stimulus onset asynchrony, SOA) was assigned randomly from 24 intervals ranging from −1500 to 1500 ms (±10, ±30, ±60, ±80, ±100, ±150, ±200, ±300, ±400, ±600, ±900, ±1500 ms) in the crossed condition, and from −750 to 750 ms (±5, ±15, ±30, ±40, ±50, ±75, ±100, ±150, ±200, ±300, ±450, and ±750 ms) in the uncrossed condition (negative and positive intervals indicate, respectively, that either the left or the right hand was stimulated first). The inter-trial intervals ranged between 2000 and 4000 ms.

Each participant underwent four experimental sessions over the course of one day. The four sessions were designed in a factorial manner so to orthogonally manipulate the arm posture (crossed vs. uncrossed) and the response strategy (earlier vs. later, see below). The order of the four sessions was counterbalanced across participants. Each session consisted of seven blocks, in each of which 24 trials were presented corresponding to a random permutation of the 24 inter-stimulus intervals. Each session thus consisted of 168 trials, for a total of 672 trials in the experiment.

Tactile stimuli were delivered by means of two miniature solenoid tappers attached to the dorsal surface of the middle fingers. The solenoids produced a supra-threshold vibrotactile stimulus oscillating at 100 Hz for a total duration of 30 ms. During the experiment, the subjects were blindfolded; moreover, white noise was presented through headphones to mask any noise. Therefore, subjects could not see or hear, but could only feel the tactile stimulation presented to the fingers.

Participants rested their right and left index fingers on two response buttons located on a table. They were asked to make two-alternative forced-choice judgments about the order of stimulation by pressing the button under the index finger of the hand they identified as being stimulated either earlier or later than the other. These two response strategies (earlier and later) were counterbalanced across the sessions in each posture condition. We used two response strategies to compensate for the participants’ bias towards reacting with the preferred hand when they are not sure about their decision^24^. Trials with response times longer than 3000 ms or without a response were re-presented at the end of the corresponding block. This procedure ensures us to have the same number of trials in all the experimental conditions. No feedback was given to the subjects.

### Data Analysis

Data analysis was as follows: first, we reversed the response data from the later response strategy and combined them with the responses in the earlier response strategy; next, for each SOA, we calculated the average order-judgment probabilities that the left (right) hand was stimulated later (earlier) in the uncrossed (*P*_*u*_) and crossed (*P*_*c*_) arm posture conditions.

Then, we fitted the individual order-judgment probabilities in the uncrossed condition (*P*_*u*_) by using a four-parameter Gaussian cumulative density function^23^ (see Supplementary Materials). According to previous studies, the individual order-judgment probabilities in the crossed condition (P_*c*_) were fitted by a five-parameter Gaussian flip model^23^ (see Supplementary Materials). This model accounts for judgment reversals caused by the arm crossing manipulation^23^. From both models, two main parameters of interest can be derived, namely the mean “d” (d_*u*_and d_*c*_, here and after the subscript *u* and *c* represent the uncrossed and crossed conditions, respectively), indicating the participants’ bias, and the standard deviation “σ” (σ_*u*_ and σ_*c*_,), indicating the participants’ precision or temporal sensitivity in the task.

Moreover, to assess increases in the judgment reversals caused by the arm crossing manipulation, we computed the so-called sum of confusions (*SC*), defined as the sum of the differences between the response functions in the crossed and uncrossed conditions (respectively, *P*_*c*_ and *P*_*u*_) using the formula described in Wada and colleagues^30^, and reported in the Supplementary Materials. We also contrasted the differences between the response functions in the uncrossed and crossed conditions across the schizotypy groups.

We carried out one-way ANOVAs to test for differences across schizotypy groups on the values of the different parameters of the fitted models as well as on the other reversal measures. Following previous studies using the Gaussian flip model (e.g.,^23^,^24^), we carried out separate one-way ANOVAs on the uncrossed and crossed condition to test for differences across schizotypy groups on the values of the parameters of the fitted models. Indeed, since the uncrossed and crossed models are quite different (and have a different number of parameters), the corresponding parameters are not easily comparable. This is true especially for the sigma parameters *σ*_*u*_ and *σ*_*c*_. Indeed, despite both indicate participants’ precision in the TOJ task, *σ*_*u*_ and *σ*_*c*_ measure the standard deviation of two different distributions (see above). Nonetheless, we directly compared the TOJ performance in the uncrossed and crossed conditions across schizotypy groups in subsequent analyses on the other reversal measures. To this aim, we performed i) a one-way ANOVA on SC across schizotypy groups (note: based on the definition of SC reported above, this analysis is mathematically equivalent to a mixed two-ways ANOVA testing for the interaction between the within-subjects posture modulation factor - uncrossed vs. crossed - and the between-subjects schizotypy group factor - low vs. moderate vs. high), and ii) a mass-univariate analysis contrasting uncrossed and crossed response functions between high and low/moderate schizotypy groups. Tukey’s honest significant difference (HSD) post-hoc tests, as implemented in Statistica analysis software (StatSoft, Tulsa, OK, USA), were performed where necessary.

## Results

### Temporal Order Judgments in the Uncrossed Condition

**Figure 1:**
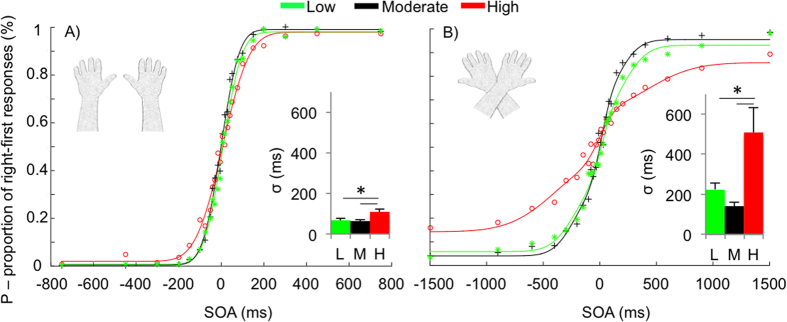
Order judgment probabilities from the uncrossed (Panel A) and crossed (Panel B) conditions for high (red), moderate (black) and low (green) schizotypy participants. The x-axis indicates stimulus onset asynchronies (SOAs) in milliseconds; the y-axis indicates the proportion of right-first responses. Negative SOAs indicate that the stimulus was delivered to the left hand first. Symbols indicate single data points. The insets represent temporal sensitivity (σ) in low (L), moderate (M) and high (H) schizotypes.

Visual inspection of the cumulative Gaussian curves shown in Fig. 1A suggests that the temporal resolution (*σ*_*u*_) was higher in the high schizotypy group as compared to the low and moderate groups. This impression was confirmed by a one-way ANOVA, which showed a significant difference in the *σ*_*u*_ values across the three schizotypy groups (*F*(2, 45) = 5.20, *p* = 0.009, *η*^2^_p_ = 0.188). The Tukey’s HSD post-hoc test revealed that the *σ*_*u*_ values were significantly higher in the high schizotypy group (109 ms, *SD* = 62 ms) as compared to both the moderate (63 ms, *SD* = 28 ms; *p* = 0.015) and low (68 ms, *SD* = 35 ms; *p* = 0.029) schizotypy groups, which did not differ from each other (*p* = 0.962).

### Temporal Order Judgments in the Crossed Condition

With arms crossed, judgment reversal was observed in all the schizotypy groups (Fig. 1B), as it is clear by visually comparing the order-judgments probability curves shown in Fig. 1A,B. Moreover, visual inspection of Fig. 1B also indicates that, in the crossed condition, the time window of the judgment reversal (*σ*_*c*_) was clearly higher in the high schizotypy group as compared to the low and moderate groups. This impression was confirmed by a one-way ANOVA, which showed a significant difference in the *σ*_*c*_ values across the three schizotypy groups (*F*(2, 45) = 6.71, *p* = 0.003, *η*^2^_p_ = 0.230). The Tukey’s HSD post-hoc test revealed that the *σ*_*u*_ values were significantly higher in the high schizotypy group (509 ms, *SD* = 495 ms) as compared to both the moderate (141 ms, *SD* = 73 ms; *p* = 0.003) and low (222 ms, *SD* = 130 ms; *p* = 0.025) schizotypy groups, which did not differ from each other (*p* = 0.720).

The one-way ANOVA on the *SC* measure, which reflects the degree of TOJ reversals caused by the arm crossing manipulation (Supplementary Materials, Eq. 7), revealed that *SC* values were significantly different among schizotypy groups *F*(2, 45) = 7.00, *p* = 0.002, *η*^2^_p_ = 0.237). The Tukey’s HSD post-hoc test revealed that the high schizotypy participants showed larger SC values (589, *SD* = 449) as compared to both the low (203, *SD* = 156; *p* = 0.003) and moderate (265, *SD* = 263; *p* = 0.015) schizotypy groups, which in turn did not differ from each other (*p* = 0.842).

### Early and late contributions to the effect of crossing the hand

Differences in hand-crossing effect on TOJ performance between groups, as showed by the results on the *SC* measure, might originate from inaccurate early somatotopic representations in high schizotypy as compared to low and moderate schizotypy participants. Alternatively, such differences might originate from a later mechanism pertaining with changing frame of reference when the hands are crossed. Indeed, earlier neurophysiological studies showed that the earliest effects of body posture on touch processing are seen reliably after 70–90 ms^31^. If the first hypothesis is true, we expect between-groups differences in the difference between the response functions in the crossed and uncrossed conditions (*P*_*c*_ − *P*_*u*_, i.e., the probability of TOJ reversals in the crossed posture with respect to the uncrossed posture) already at early delays (SOAs < 100 ms). If the second hypothesis is true, we expect between groups differences only at later delays (SOA > 100 ms).

**Figure 2:**
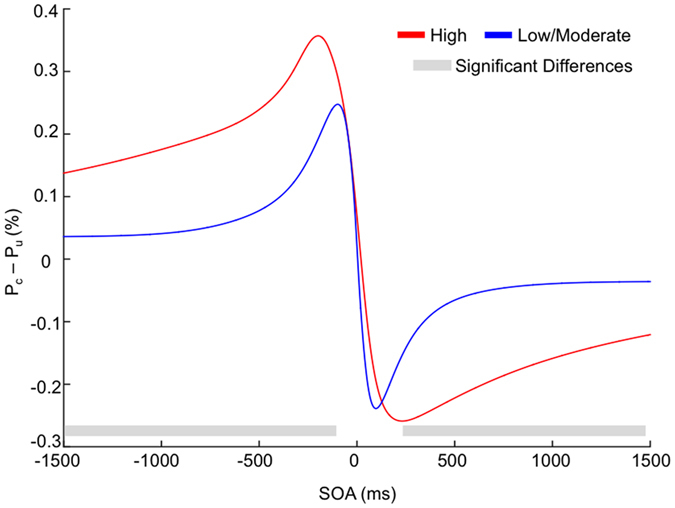
Difference in the order judgment probabilities between the uncrossed and the crossed-hand posture (P_c_ − P_u_) in the high (Red) and low/moderate (Blue) schizotypy participants. The x-axis indicates stimulus onset asynchronies (SOAs) in milliseconds Grey areas represent significant differences.

To disentangle between these two alternatives, we compared the *P*_*c*_ − *P*_*u*_ difference across groups in every single delay (i.e., for every 1 ms in the −1500–1500 ms range) with a series of two-tailed independent-samples *t*-test followed by a cluster-based permutation test to control for multiple comparisons^32^. As the low and moderate schizotypy participants did not differ from each other in any of the previous analyses, the two groups were collapsed. The analysis revealed that the probability of TOJ reversals in the crossed posture was significantly higher in high schizotypy participants as compared to low/moderate schizotypy participants at long delays, specifically, in the time windows from −1500 to −135 ms and from 234 to 1500 ms (see Fig. 2).

### Correlations between *SC* and SPQ subscales

**Figure 3:**
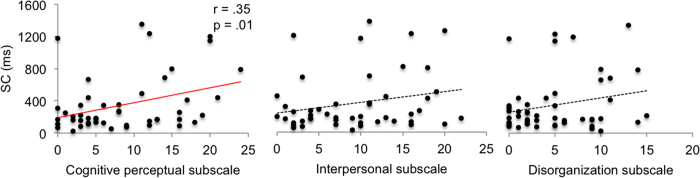
Correlations between the three SPQ subscales and sum of confusion (SC) scores.

Inspecting the exact relationship between SC (reflecting the degree of TOJ reversals caused by the arm crossing manipulation) and each dimension of the schizotypy construct is crucial for understanding whether and to what extent abnormal perceptual experiences, abnormal social interactions and disorganized behavior could be predicted by our task. With the aim of clarifying this issue, we carried out correlation analyses between the three SPQ subscales and SC scores (see Fig. 3). Results (Bonferroni corrected, *p* < 0.017) revealed a positive correlation between the cognitive-perceptual subscale of the SPQ and SC scores (*r* = 0.35, *p* = 0.014, CI = [0.06 0.61]), while the correlations with the Interpersonal (*r* = 0.23, *p* = 0.11, CI = [−0.034 0.49]) and the Disorganization (*r* = 0.23, *p* = 0.12, CI = [−0.049 0.52]) subscales were not significant.

## Discussion

The present study aimed to evaluate the contribution of somatosensory dysfunction and abnormal remapping of touch to schizotypal personality traits. To this aim, we sampled 48 participants with low, moderate and high schizotypy from a population of 411 and compared their performance during crossed and uncrossed TOJ tasks. First, the temporal resolution (*σ*_*u*_) in high schizotypy participants was significantly worse in the uncrossed task, compared to low and moderate schizotypy participants. This was likely due to known abnormalities in tactile and proprioceptive perception in schizotypy^4^,^5^. Indeed, body-centered somatosensory information provides the main contributions during the uncrossed TOJ task^33^. Second, the time window of the order-judgments flip (*σ*_*c*_) in the crossed task was significantly larger in high schizotypy participants than it was in both low and moderate schizotypy participants. Third, we demonstrated that possible somatosensation deficits in high schizotypy could not fully explain the abnormal remapping of touch during the crossed TOJ. Indeed, the high schizotypy, compared to moderate and low schizotypy, showed higher degree of TOJ reversals caused by the arm crossing manipulation as assessed by the *SC*), a measure that takes into account the individual variability in the uncrossed TOJ performance. Fourth, the higher probability of TOJ reversals in the crossed posture in high schizotypy, compared to all the other participants, emerged reliably at later SOAs (>100 ms), that is, when tactile remapping has already started^31^. This result suggests that deficits in early somatosensory processing cannot fully account for the strong crossed-hand effect in high schizotypy. Fifth, the results showed that the differences in TOJ performance in high schizotypy and their stronger crossed-hand effect cannot be explained by possible, unspecific effects such as the degree of general errors in reporting left-first and right-first stimuli in the uncrossed condition, or the baseline error rate (*c*) in the crossed condition (see Supplementary Materials for details).

Why is the crossed–hand effect stronger in schizotypy? To approach this question, it is worth considering the theoretical perspectives that have been proposed to account for the TOJ crossed-hand effect in healthy participants. Kitazawa and colleagues^23^,^34^ suggested that tactile events are always encoded to an external frame of reference, and touch remapping is directed from the external to an anatomical location. According to this assumption, the crossed-hand effect would be due to a conflict between the usual and current location of the touch in external spatial coordinates. Instead, Shore and colleagues^24^,^35^ proposed that a tactile stimulus is initially represented according to its anatomical location on the skin and then remapped into external coordinates. According to this alternative view, the crossed-hand effect would be due to the concurrent availability of the two spatial representations, each based on a different reference frame. Although different in many ways, both accounts posit that the crossed-hand effect reveals that touch remapping engages a system of multiple reference frames characterized by multisensory input. Specifically, tactile cues and proprioceptive information about the current body posture are integrated with exteroceptive information, which are possibly based on representations of visual space (i.e., the space mapped according to retinal coordinates). Hence, somatosensory processing and multisensory integration are both essential for touch remapping.

These functions actually play a major role already in the uncrossed TOJ task^33^. However, our data suggest that group differences in early somatosensory processing and integration of tactile and proprioceptive information cannot fully explain the stronger crossed-hand effect in high, compared to moderate and low schizotypy. Rather, the stronger crossed-hand effect in high schizotypy seems to be explained by alterations in subsequent touch remapping processes involving the integration of tactile and proprioceptive cues with external spatial information, as revealed by the analysis on SC. Are we, then, allowed to rule out any contributions of altered tactile and proprioceptive processing to this result? Or, should we look into the possibility that the deficit in the integration of anatomical (i.e., tactile and proprioceptive) and external spatial information magnifies the effects of altered somatosensation in high schizotypy? This hypothesis would be corroborated by the fact that, as any other multisensory integration processes, the integration of different types of spatial information is not fixed, but flexibly adjusted according to the context^33^ and the reliability of sensory information^36^. As a consequence, the weighting of information from different sources is such that the importance given to a source is closely related to its reliability^37^. For example, haptic information is regarded more when the quality of visual information is degraded^38^. According to this evidence, altered reliance in somatosensation should affect the integration of anatomical and external spatial information. This is indeed the case in autistic children. They are characterized by greater reliance on proprioceptive information^39^, and show a significantly smaller crossed-hand effect than neurotypical children^30^. Hence, the greater reliance in proprioception of children with autism generates a bias in the weighting of anatomical and external spatial information during the TOJ crossed-hand task. Conversely, our results on high schizotypy could be explained by the fact that a smaller reliance in altered somatosensory information might lead to assign higher weight to external, than anatomical, spatial information. As a result, and contrary to what observed in autism, people with high schizotypy show stronger, rather than smaller, crossed-hand effect, when compared to people with moderate and low schizotypy. This evidence clearly illustrates how multisensory integration abnormalities, which are common to several clinical conditions (e.g.,^40^,^41^,^42^), might result in opposite symptoms. Furthermore, it adds to previous knowledge of multisensory integration deficits, as it reveals that abnormal integration of anatomical and external spatial information possibly contributes to psychosis risk.

### Spatial remapping of touch and self boundaries in schizotypy

Recently, Postmes^43^ and colleagues suggested a potential relationship between multisensory integration deficit in schizophrenia spectrum disorders^9^,^27^,^28^,^44^,^45^,^46^,^47^ and incoherent experience of self and self-environment boundaries^48^,^49^,^50^,^51^,^52^. Such proposal is grounded on evidence that the development of a sense of self, and of its demarcation from the environment, is intertwined with the developmental abilities to correctly integrate sensory input^53^,^54^. In particular, when the input of different sensory modalities is merged, the ability to correctly identify and localize their sources is essential to distinguish between self and environment. Assuming a dynamic continuum from schizotypy to schizophrenia^14^, we can speculate that abnormal integration of spatial information in schizotypy has to do with the proposed effect of altered multisensory integration on the demarcation of self boundary in schizophrenia. This idea is supported by empirical studies in schizotypy and schizophrenia showing similar results^14^.

There is plenty of evidence showing that self-environment discrimination and sense of boundaries is enabled by proprioception and touch^55^,^56^,^57^. Accordingly, reduced proprioceptive or haptic sense may lead to diminished boundary recognition, as suggested by increased proneness to the RHI in schizotypy^8^ and schizophrenia^8^,^58^,^59^. In general, the stronger proneness to the illusion reveals more blurred boundaries between self-body and the environment. In schizotypy and schizophrenia this was demonstrated not only by self-report questionnaires, but also by proprioceptive drift – i.e., an erroneous localization of the participant’s touched hand toward the rubber hand. These results indirectly support the idea that the integration of body-centred and external spatial information is altered in schizotypy and schizophrenia. Our study provides more direct evidence for this idea.

In sum, for the first time, we directly and specifically revealed that the coordinate transformation between different reference frames, an essential function to demarcate the boundaries between self and environment, is altered in schizotypy. Thus, such phenotype may represent a behavioral risk marker for schizophrenia, likely associated to altered spontaneous oscillation at the neural level. Indeed, Heed and colleagues^2^ recently suggested that oscillatory brain activity seems to be optimally suited for implementing the transformation and integration of multiple coordinates. Interestingly, there is now plenty of evidence associating altered spontaneous brain oscillations with risk for psychosis^60^,^61^,^62^,^63^,^64^, as well as abnormal multisensory perception in schizophrenia^65^,^66^. Future research is warranted to determine a direct relationship between abnormal touch remapping and altered oscillatory brain activity in schizotypy.

## Additional Information

**How to cite this article:** Ferri, F. *et al*. Spatiotemporal processing of somatosensory stimuli in schizotypy. *Sci. Rep.* **6**, 38735; doi: 10.1038/srep38735 (2016).

**Publisher's note:** Springer Nature remains neutral with regard to jurisdictional claims in published maps and institutional affiliations.

## Supplementary Material

Supplementary Materials

## Figures and Tables

**Table 1:** Demographics and SPQ scores of the participants included in the current study.

	N	No. of Female	Mean Age	Mean SPQ Score	SPQ Range
Low schizotypes	16	9	24.9	6.2	0–11
Moderate schizotypes	16	9	24.1	19.6	17–22
High schizotypes	16	9	23.5	36.7	31–47
