# Supplementary material for: Spatiotemporal processing of somatosensory stimuli in schizotypy
Source: Sci Rep. 2016 Dec 9;6:38735. doi: 10.1038/srep38735 (PMC5146666; doi:10.1038/srep38735)
Supplement: Supplementary Materials [file srep38735-s1.doc]

# Spatiotemporal processing of somatosensory stimuli in schizotypy

Francesca Ferri^1^, Ettore Ambrosini^2^ & Marcello Costantini^1,3^

^1^ Centre for Brain Science, Department of Psychology, University of Essex, Colchester, UK

^2^ University of Padova

^3^ Laboratory of Neuropsychology and Cognitive Neuroscience, Department of Neuroscience and Imaging, University G. d’Annunzio, Chieti, Italy & Institute for Advanced Biomedical Technologies ‐ ITAB, Foundation University G. d’Annunzio, Chieti, Italy.

**SUPPLEMENTARY MATERIALS**

**Data analysis**

Four hundred eleven students were screened with respect to schizotypal traits using the Schizotypal Personality Questionnaire[^16^](#_ENREF_16). The distribution of scores (see Supplementary Figure S1 and Supplementary Table T1) was divided into quintile, with fifth quintile representing the students rated as high schizotypes, the first quintile representing the students rated as low schizotypes, and the third quintile representing the student rated as moderate schizotypes. The second and fourth quintiles were not used.

**
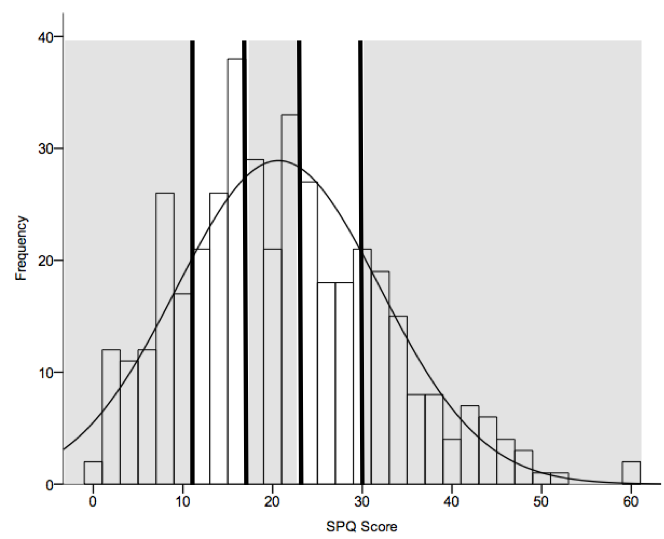
**

**Figure S1:** Distribution of SPQ scores across the screened students. From left to right, grey areas represent the first, third and fifth quintile respectively.

| **Percentile Group of SPQ** |  |  | |  |  |
| --- | --- | --- | --- | --- | --- |
|  | **N** | **Min** | **Max** | **Mean** | **SD** |
| **1st Quintile** | **80** | **0** | **11** | **6.0** | **2.9** |
| 2nd Quintile | 85 | 12 | 16 | 13.9 | 1.8 |
| **3rd Quintile** | **83** | **17** | **22** | **19.6** | **1.7** |
| 4th Quintile | 84 | 23 | 30 | 26.3 | 2.4 |
| **5th Quintile** | **79** | **31** | **62** | **37.9** | **6.9** |

**Table T1:** Distribution of SPQ scores across the screened students. Bold represents the quintile considered in the current study.

*Uncrossed condition - Gaussian distribution*

Data analysis was as follows: first, the response data from the later response strategy were reversed and combined with the responses in the earlier response strategy to calculate, for each SOA, the average order-judgment probabilities that the right hand was stimulated earlier (or the left hand was stimulated later) in the uncrossed (*P_u_*) and crossed (*P_c_*) arm posture conditions.

Then, the individual order-judgment probabilities in the uncrossed condition (*P_u_*) were fitted by a following four-parameter cumulative density function of a Gaussian distribution (Yamamoto & Kitazawa, 2001):

**Equation 1:**
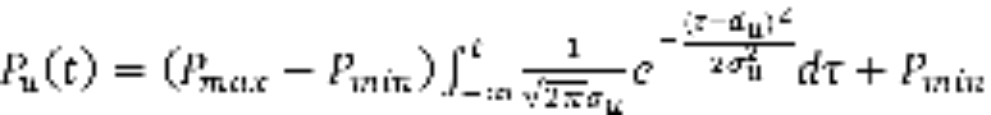


In this equation, *t* indicates the stimulation interval, the two parameters *P_min_* and *P_max_* denote, respectively, the lower and upper asymptote to which the cumulative Gaussian function was limited, and the other two parameters *d_u_* and *σ_u_* denote the mean and the standard deviation of the Gaussian distribution, respectively. Therefore, the parameter *d_u_* is a measure of bias that corresponds to the so-called point of subjective simultaneity between the two stimuli (or size of the horizontal transition), whereas the parameter *σ_u_* indicates the participants’ precision in the TOJ task, that is, the just noticeable difference in time between two stimuli. This index provides a standardized measure of the sensitivity with which participants can correctly judge the temporal order of the two tactile stimuli with 84% of accuracy. Note also that it is independent of the general error in judgments that is reflected by the *P_min_* and *P_max_* parameters; it rather indicates the time window of uncertainty in temporal order judgments, or their temporal resolution. The Matlab Optimization toolbox (The Mathworks, Natick, MA) was used to minimize the Pearson’s *χ*^2^ statistic, which reflects the discrepancy between the observed order-judgment probabilities (24 data points) and the prediction obtained by using the four-parameter cumulative Gaussian model (Eq. 1).

*Crossed-hand condition - Gaussian flip model*

To evaluate whether the participant’s temporal order judgments in the crossed condition (*P_c_*) significantly deviated from those in the uncrossed condition, we tested the null hypothesis that the data could be represented by a cumulative Gaussian model (Eq. 1) with the parameters for bias *d* and precision *σ_u_* (or only the precision parameter *σ_u_*) were fixed to be the same as those found in the uncrossed condition (Moizumi, Yamamoto, & Kitazawa, 2007). A significant (*p* < .05) goodness-of-fit test using Pearson’s *χ*^2^ statistic (*df* = 19) indicates that the participant’s temporal order judgments in the crossed condition were not reliably fitted by the cumulative Gaussian function used in the uncrossed condition and, thus, that they were altered by the postural manipulation. In any case, we also fitted the individual order-judgment probability in the crossed condition (*P_c_*) by optimizing all of the four parameters of the cumulative density function of a Gaussian distribution as in Eq. 1.

If the participants’ judgments in the crossed condition were found to be altered, it can be hypothesized that the corresponding temporal order-judgment probability under the crossed condition (*P_c_*) was flipped (i.e., reversed) from the order-judgment probability in the uncrossed condition (*P_u_*) as predicted by the Gaussian flip model (Yamamoto & Kitazawa, 2001):

**Equation 2:**
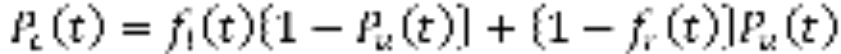


**Equation 3:**
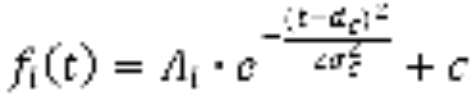


**Equation 4:**
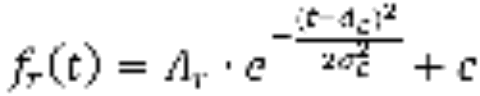


Here, *f_l_* indicates the flip probability of judgment from “left first” to “right first” (i.e., the probability that the correct “left first” judgments were reversed), whereas *f_r_* indicates the flip probability of judgment from “right first” to “left first” (i.e., the probability that the correct “right first” judgments were reversed). Therefore, the Gaussian flip model is defined by five parameters as shown in Eq. 3-4: the two parameters *A_l_* and *A_r_*, which denote the peak flip amplitudes of the Gaussian functions; the two parameters *d_c_* and *σ_c_*, which denote the mean and the standard deviation of the Gaussian flip distributions, respectively; and a parameter *c* for the constant. The parameters *A_l_* and *A_r_* indicate the tendency to make judgment reversal at short stimulation intervals (i.e., SOAs), which subsides at longer intervals, whereas the parameter *c* indicates the probability of a generic error in the judgments. The parameter *σ_c_* indicates the time window of the flip and, thus, it reflects the uncertainty in temporal order judgments. Again, the Matlab Optimization toolbox was used for estimations to minimize the Pearson’s *χ*^2^ statistic. To assess whether the five-parameter Gaussian flip model provide a better explanation of the participants’ performance in the crossed condition as compared to the four-parameter cumulative Gaussian model, we carried out *χ*^2^ difference tests.

Following Wada et al. (Wada, Yamamoto, & Kitazawa, 2004), we also calculated net peak flip amplitudes (*Ã_l_* and *Ã_r_*) as follows:

**Equation 5:**
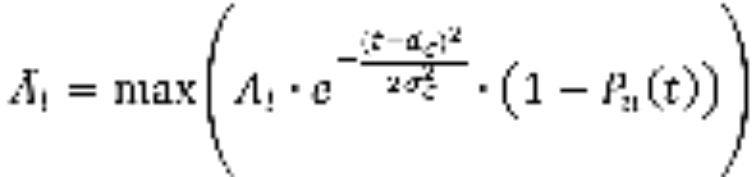


**Equation 6:**
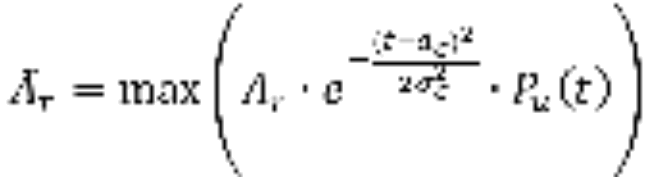


*Sum of confusions*

To assess increases in the judgment reversals caused by the arm crossing manipulation, we computed the so-called sum of confusions (*SC*), defined as the sum of the differences between the response functions in the crossed and uncrossed conditions (respectively, *P_c_* and *P_u_*) using the following formula:

**Equation 7:**
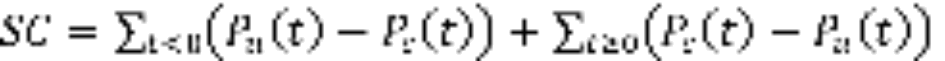


# Results

## *Temporal Order Judgments in the Uncrossed Condition*

When the participants’ arms were uncrossed, the order-judgment probability (*P_u_*) that the right hand was stimulated first was well approximated by the cumulative Gaussian function (Eq. 1) in the low, moderate, and high schizotypes (Fig. 1A). Indeed, the *χ*^2^ goodness-of-fit tests revealed that the cumulative Gaussian model was not rejected in any participant in the low (all *χ*^2^(19) < 28.8, *p*s > .069) and moderate schizotypy groups (all *χ*^2^(19) < 25.7, *p*s > .139), although it was rejected in two out of the 16 participants in the high schizotypy group. Moreover, the mean *r*^2^ values for the low, moderate, and high schizotypy groups were, respectively, .950 (range: .885-.973), .953 (range: .917-.988), and .913 (range: .769-.983). A one-way ANOVA revealed a significant difference between the *r*^2^ values of the three participant groups (*F*(2, 45) = 4.67, *p* = .014, *η*^2^_p_ = .172), and a Tukey’s HSD post-hoc test showed that they were significantly lower in the high schizotypy group as compared to both the moderate (*p* = .022) and low (*p* = .041) schizotypy groups, which did not differ from each other (*p* = .965).

*One-way ANOVA on the parameter d_u_*

The one-way ANOVA on the parameter *d_u_* revealed that the size of the horizontal transition, corresponding to the point of subjective simultaneity, did not significantly differ across groups (*F*(2, 45) = .71, *p* = .495, *η*^2^_p_ = .031). Moreover, the mean *d_u_* value was not significantly different from zero both in the whole sample (*F*(2, 45) = .58, *p* = .450, *η*^2^_p_ = .013) and in any of the three schizotypy groups (low: < 0 ms, *SD* = 36 ms, *t*(15) = .04, *p* = .967, Cohen’s *d* = .01; moderate: -1 ms, *SD* = 15 ms, *t*(15) = -.27, *p* = .790, *d* = -.07; high: 10 ms, *SD* = 33 ms, *t*(15) = 1.28, *p* = .221, *d* = .32).

*One-way ANOVA P_min_ and P_max_*

The one-way ANOVA on the parameters *P_min_ and P_max_* denoting, respectively, the lower and upper asymptotes of the cumulative Gaussian curves revealed no significant differences across the three schizotypy groups (respectively, *F*(2, 45) = .57 and .70, *p* = .571 and .506, *η*^2^_p_ = .025 and .030). Moreover, the *P_min_* and (1 – *P_max_*) values (which reflect the general error in reporting left-first and right-first stimuli, respectively) did not differ from each other in any of the schizotypy groups (all *t*s(15) < .65, *p*s > .525).

## *Temporal Order Judgments in the Crossed Condition*

The *χ*^2^ goodness-of-fit tests revealed that, when the participants’ arms were crossed, their TOJ performance was not accurately explained by a cumulative Gaussian model (Eq. 1) with the same bias and precision parameters *d* and *σ_u_* as those in the uncrossed condition. Indeed, such a cumulative Gaussian model was rejected in all of the participant in the high (all *χ*^2^(19) > 67.1, *p*s < .001) and low schizotypy groups (all *χ*^2^(19) < 40.7, *p*s > .001), and in all but two participants in the moderate schizotypy group. Similarly, a cumulative Gaussian model with the same precision parameter *σ_u_* as that in the uncrossed condition was rejected in the vast majority of the participants (respectively, 16/16, 13/16, and 12/16 of the participants in the high, low, and moderate schizotypy groups. Finally, a cumulative Gaussian model in which all of the four parameters were optimized was rejected in the majority (i.e., 10/16) of the high schizotypy participants and in 3/16 and 6/16 of the participants in the low and moderate schizotypy groups. Interestingly, a *χ*^2^ test revealed that the distribution of these proportions across groups was not due to chance (*χ*^2^(4) = 6.45, *p* = .040) due to the higher proportion of high schizotypy participants whose TOJ performance could not be adequately fitted by the cumulative Gaussian function.

These analyses thus revealed that the participants’ judgments were significantly altered by the crossed arms manipulation as compared to the uncrossed condition. We thus fitted the corresponding temporal order-judgment probability in the crossed conditions (*P_c_*) by using the Gaussian flip model (Yamamoto & Kitazawa, 2001). The order-judgment probability that the right hand was stimulated first in the crossed condition (*P_c_*) was well approximated by the Gaussian flip model (Eq. 2-4) in the low, moderate, and high schizotypy groups (Fig. 1B). Indeed, the *χ*^2^ goodness-of-fit tests revealed that the cumulative Gaussian model was not rejected in all of the participants in the low schizotypy group and in all but one participant in both the high and moderate schizotypy groups. Moreover, the *χ*^2^ difference tests showed that the five-parameter Gaussian flip model provide a better explanation of the participants’ performance in the crossed condition, as compared to the four-parameter cumulative Gaussian model, in the majority of participants in the high, moderate, and low schizotypy groups (i.e., 12/16, 10/16 and 9/16, respectively), as well as in the three groups as a whole (respectively, *χ*^2^(1) = 10.84, 33.68, and 47.28; all *p*s < .001). Furthermore, the mean *r*^2^ values for the high, moderate, and low schizotypy groups were, respectively, .754 (range: .266-.960), .930 (range: .787-.989), and .920 (range: .819-.983). Confirming the pattern found for the uncrossed condition, a one-way ANOVA revealed a significant difference between the *r*^2^ values of the three participant groups (*F*(2, 45) = 9.23, *p* < .001, *η*^2^_p_ = .291), and a Tukey’s HSD post-hoc test showed that they were significantly lower in the high schizotypy group as compared to both the moderate (*p* = .001) and low (*p* = .002) schizotypy groups, which did not differ from each other (*p* = .973).

*One-way ANOVA on d_c_*

The one-way ANOVA carried out on the *d_c_* values did not reveal significant differences across schizotypy groups (*F*(2, 45) = .90, *p* = .414, *η*^2^_p_ = .038). Moreover, the mean *d_c_* value was not significantly different from zero both in the whole sample (*F*(2, 45) = .98, *p* = .328, *η*^2^_p_ = .021) and in any of the three schizotypy groups (low: 12 ms, *SD* = 112 ms, *t*(15) = .42, *p* = .683, Cohen’s *d* = .10; moderate: -17 ms, *SD* = 96 ms, *t*(15) = -.70, *p* = .495, *d* = -.17; high: -61 ms, *SD* = 222 ms, *t*(15) = -1.09, *p* = .291, *d* = -.27).

*One-way ANOVA on c*

One-way ANOVA on the parameter *c*, which indicates the probability of a generic error in the judgments. This analysis did not reveal significant differences across schizotypy groups (*F*(2, 45) = 1.33, *p* = .276, *η*^2^_p_ = .056).

*One-way ANOVAs on Ã_l_* and *Ã_r_*

The one-way ANOVAs on the participants’ net peak flip amplitudes *Ã_l_* and *Ã_r_* (which represent, respectively, the maximum probability of TOJ reversal for right-first and left-first stimuli that were added to the baseline error rate *c*, see Supplementary figure 2 and Eq. 5-6) revealed that these parameters were not significantly different among the three schizotypy groups (respectively, *F*(2, 45) = 1.42 and .62, *p* = .253 and .543, *η*^2^_p_ = .059 and .027).

**
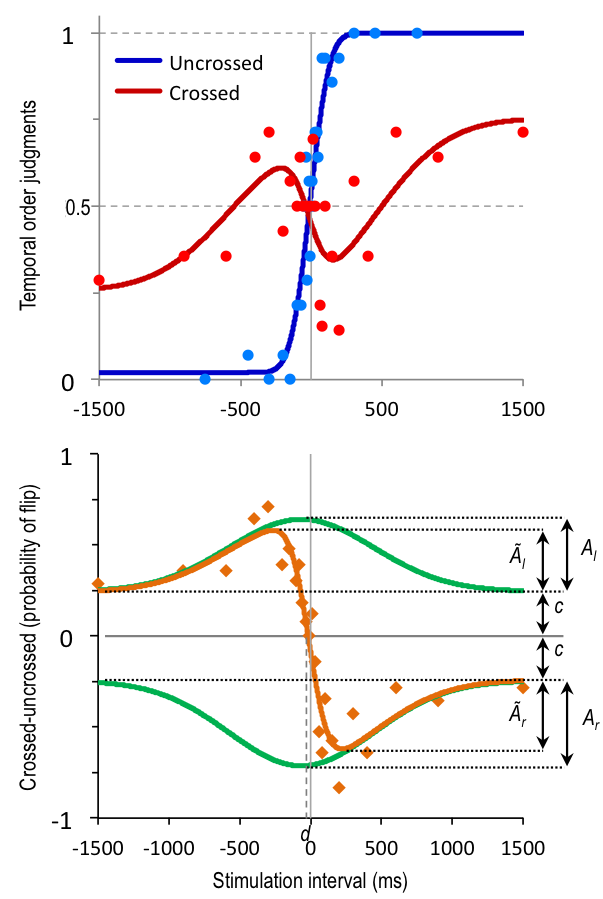
**

**Supplementary Figure S2:** TOJ performance in a participant showing the characteristic N-shaped response curves in the crossed condition. The upper panel shows the proportion of right-first (or left-second) judgments (ordinate) plotted against the stimulation interval (abscissa). The blue dots and curve show, respectively, the responses and the cumulative Gaussian fit in the uncrossed condition. The red dots and curve show, respectively, the responses and the Gaussian Flip fit in the crossed condition. In the lower panel, the difference between the order-judgment responses and probabilities (orange dots and curve, respectively) in the crossed and uncrossed condition (ordinate) is plotted against the stimulation interval (abscissa). The upward and downward Gaussian curves (green) represent, respectively, the *f_l_* and *f_r_* Gaussian flip functions as defined in Eq. 3-4. The figure also indicates the mean of the Gaussian flip distribution, the probability of generic error (*c*) and the peak flip amplitudes (*A_l_* and *A_r_*) from Eq. 3-4, as well as the net peak flip amplitudes (*Ã_l_* and *Ã_r_*) from Eq. 5-6.

Overall, supplementary analyses showed that the differences in TOJ performance in high schizotypy and their stronger crossed hand effect cannot be explained by possible, unspecific effects such as the degree of general errors in reporting left-first and right-first stimuli (*P_min_* and *P_max_*) in the uncrossed condition or the baseline error rate (*c*) in the crossed condition.

**Supplementary References**

Moizumi, S., Yamamoto, S., & Kitazawa, S. (2007). Referral of tactile stimuli to action points in virtual reality with reaction force. *Neurosci Res, 59*, 60-67.

Wada, M., Yamamoto, S., & Kitazawa, S. (2004). Effects of handedness on tactile temporal order judgment. *Neuropsychologia, 42*, 1887-1895.

Yamamoto, S., & Kitazawa, S. (2001). Reversal of subjective temporal order due to arm crossing. *Nat Neurosci, 4*, 759-765.
